# On-Line Adaptive Radiation Therapy: Feasibility and Clinical Study

**DOI:** 10.1155/2010/407236

**Published:** 2010-11-22

**Authors:** Taoran Li, Xiaofeng Zhu, Danthai Thongphiew, W. Robert Lee, Zeljko Vujaskovic, Qiuwen Wu, Fang-Fang Yin, Q. Jackie Wu

**Affiliations:** ^1^Department of Radiation Oncology, Duke University Medical Center, Durham, NC 27710, USA; ^2^Department of Radiation Oncology, The Brody School of Medicine, East Carolina University, Greenville, NC 27834, USA

## Abstract

The purpose of this paper is to evaluate the feasibility and clinical dosimetric benefit of an on-line, that is, with the patient in the treatment position, Adaptive Radiation Therapy (ART) system for prostate cancer treatment based on daily cone-beam CT imaging and fast volumetric reoptimization of treatment plans. A fast intensity-modulated radiotherapy (IMRT) plan reoptimization algorithm is implemented and evaluated with clinical cases. The quality of these adapted plans is compared to the corresponding new plans generated by an experienced planner using a commercial treatment planning system and also evaluated by an in-house developed tool estimating achievable dose-volume histograms (DVHs) based on a database of existing treatment plans. In addition, a clinical implementation scheme for ART is designed and evaluated using clinical cases for its dosimetric qualities and efficiency.

## 1. Introduction

**Figure 1:**
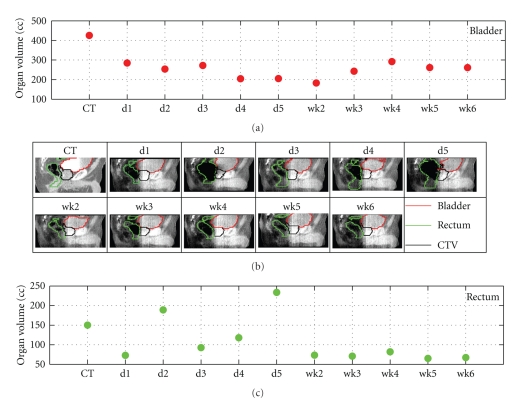
Daily structure variations of one patient due to bladder and rectum fillings. Shown in the figure are 11 images including 1 planning CT and 10 cone-beam CT (CBCT) images taken once per day for the first 5 days of treatment (d1–d5), and once per week thereafter (w2–w6). Colored contours: red—bladder, green—rectum, black—CTV.

During external beam radiation therapy (EBRT) for prostate cancer patients, interfractional anatomical variations often occur due to organ motion and/or deformation. Before the emergence of on-board 3D imaging techniques, these variations were accounted for by adding margins to the clinical target volume (CTV) to generate the planning target volume (PTV) [1, 2]. However, the large deformation/shift of organs around the prostate and the seminal vesicles can still lead to incomplete coverage of the target.  Figure 1 shows one such example, due to shape and volume variations of the bladder and the rectum, the daily CTVs (prostate and seminal vesicles) exhibit not only the position variations, but also shape changes, which lead to mismatch between high-dose region and the daily CTV.

To improve daily CTV coverage, Image-Guided Radiation Therapy (IGRT) has been widely implemented in clinical practice in recent years. For example, at our institution, in-room imaging systems such as On-Board Imager (OBI, Varian Medical Systems, Palo Alto, CA) are used to guide the on-line patient positioning as well as target alignment. The patient/target position correction schemes include 2D-2D matching, where on-board orthogonal megavoltage (MV)/kilovoltage (kV) images are matched with digitally reconstructed radiographs (DRRs) from planning CT data [3–5], and 2D-3D matching, where planning CT is shifted/rotated in three dimensions to match DRRs with daily MV/kV images [5–8]. The on-board cone-beam CT (CBCT) imaging has also led to wide implementation of 3D soft tissue target matching and repositioning. Such matching is based on daily contours [9, 10], or grey-scale values of the on-board and planning 3D images [11–14]. In most clinical protocols, soft-tissue matching is limited to translational position corrections due to restricted dimensions on couch motion.

To further improve daily dose delivery accuracy, on-line and off-line plan adaptation or modification utilizing daily volumetric imaging has been actively studied by several groups [13–27]. Plan adaptation can account for not only target position variation, but also the deformation of target and/or organ-at-risk (OAR) volumes, which occur frequently during prostate cancer treatment [5]. Some groups have proposed modification of the multileaf collimator (MLC) segments (aperture shape) directly based on daily structure shapes and positions [13–17, 19, 20]; while others have investigated off-line replanning techniques based on patient-specific target shape/volume variation trends [21–25, 28]. We have recently developed an on-line plan reoptimization technique that can correct large anatomical variations and generate highly conformal daily plans using linear programming models [5, 18].

In this paper, we summarize our approach towards a clinically applicable on-line adaptive radiation therapy (ART) system. The paper is divided into four sections. First, we present the online plan adaptation technique and demonstrate that the adaptation can be implemented with speed comparable to the repositioning-based IGRT techniques. Second, the dosimetric benefits of this on-line ART technique are evaluated against repositioning-based IGRT techniques with retrospective clinical patient case studies. Further, plan quality of the adapted plans is compared with standard IMRT plans of the same anatomy. A dose-volume histogram (DVH) evaluation tool is also developed for assessment of the ART plan quality. The actual DVH is assessed by comparing to predicted achievable DVH based on daily anatomical structures and the *learned *anatomy-DVH correlations from 198 existing treatment plans. Finally, a clinical implementation scheme is illustrated to maximize the efficiency of the ART strategy by combining both soft-tissue target matching for simple target position variation and on-line reoptimization techniques for large anatomical variations.

## 2. Methods and Materials

### 2.1. Fast Reoptimization Algorithm: Technical Feasibility

Developing a fast replanning algorithm is the first step for an adaptive radiation therapy program. Recently, several groups have proposed different on-line plan adaptation methods. They can be categorized into two groups: (1) for fluence map-based IMRT plans, modifying MLC leaf positions directly based on changes in anatomical structure positions and shapes [13, 15, 16] and (2) for direct aperture optimization- (DAO-) based plans, modifying the aperture shapes and weights using the projection of the target-organ deformation information [14, 20]. These methods feature fast adaptation but are based on manipulating either 2D fluence maps or discrete 2D beam apertures in the attempt to approximate the 3D target-OAR geometry variation and regain the 3D dose conformality of the original plan.

**Figure 2:**
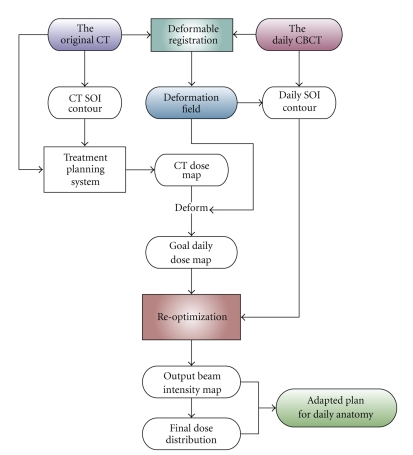
On-line plan adaptation process. After daily CBCT image sets are acquired, they are registered with planning CT image set via deformable registration, from which a 3D deformation field is generated and is used to deform original dose distribution. The deformed dose distribution is then fed to the optimization engine as the goal. In less than two minutes, reoptimized beam intensity maps are generated and final dose is calculated.

Our approach is to directly reoptimize the fluence map based on the initial IMRT plan (dose distribution) [5, 18]. This task is divided into the following steps. First, after the daily image is acquired, deformation fields are computed from deformable registration between daily CBCT and planning CT images. The original dose distribution is then deformed via the deformation fields to provide the goal dose distribution for the daily anatomy. Structures of interest (SOI) are contoured by the same attending physician who contours on the planning CT images or propagated from planning CT to CBCT via deformation fields. In the early stages of the ART implementation, CBCT image sets with physician-contoured structures have been used in our initial studies. The deformed dose is visually checked for shape irregularities and excessively high/low dose spots before being sent to the reoptimization process [5, 18]. A linear programming model is chosen for its speed and robustness for the fluence map reoptimization. The aim is to generate new fluence maps that deliver goal dose distribution. Once these new intensity maps are generated, dose distributions are recalculated and dose-volume histograms (DVHs) are computed and displayed for plan evaluation.  Figure 2 illustrates the flow of the on-line plan adaptation process.

### 2.2. Comparison of ART Plans against Repositioned Plans: Dosimetric Benefits

To evaluate the dosimetric benefits of the online ART technique, a clinical study is performed to compare this technique with current repositioning-based IGRT techniques. This retrospective study includes 6 prostate patient cases, each with 1 planning CT and 5 daily CBCT image sets. For all image sets, SOI including CTV (prostate + SV) and OARs (bladder and rectum) are contoured by a single attending physician at our institution. The urethra is not contoured in this study since it is difficult to spare in EBRT and is generally not included in the dosimetric constraints. The PTV is expanded with 5 mm uniform margin from CTV for all plans. The soft-tissue-matching-based repositioning plans (Soft) are performed by trying to cover the daily CTV with the initial planning PTV, simulating standard clinical practice of soft-tissue matching IGRT. The on-line ART (Adapt) plans are generated using our in-house developed reoptimization technique [18]. This plan optimization process is finished within two minutes for each of the 30 CBCT image sets. DVHs for all SOI are computed for both techniques. Dosimetric comparisons of the Adapt versus Soft plans are performed.

### 2.3. Plan Quality Evaluation for Adaptive Radiation Therapy (ART)

To evaluate the ART plan quality, two methods are used to assess the dose distributions to the CTVs and the OARs. One way of evaluating plan quality is to perform head-to-head comparison between an adapted plan and a complete new plan designed in the clinical environment for the same anatomy. This is the step we have taken during the initial implementation of the online ART technique. For each Adapt plan described in section 2b, a complete new plan (New) is generated using the same CBCT image and structure set. This new plan is performed with the clinical treatment planning system (Eclipse) and is used as the control sample of IMRT planning. The minimum dose to the hottest 99% of the CTV volume (D99) and the maximal dose to CTV (D_max_) are used to compare the Adapt plan quality to the New plan for target coverage. Minimum doses to the hottest 90% (D90), 50% (D50), and 30% (D30) of the OAR volumes are chosen as comparison parameters for OAR sparing, which represent dose sparing in low-, median-, and high-dose ranges, respectively. 

**Figure 3:**
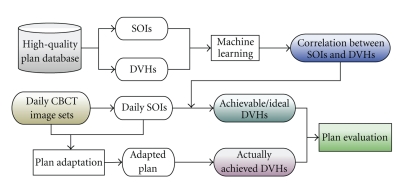
Flowchart of the quality evaluation tool for the adapted plans. DVHs and SOIs of previous *expert* plans are used to train the algorithm to establish the correlation between these two inputs. Once such correlation is *learned*, achievable/ideal DVHs can be predicted based on daily SOIs and are compared against actually achieved DVHs.

Although head-to-head comparison provides intuitive and direct assessment based on clinical standards, it is time consuming. Furthermore, the individual new plan performed in clinical planning system (Eclipse) uses a trial-and-error iterative optimization scheme, thus the plan quality would be dependent of planner's experience and time invested and should be performed by expert planners. To provide standardized plan quality assessment for ART implementation, a plan evaluation tool is developed to predict the achievable/ideal dose volume histograms (DVHs) based on patient anatomy information and the learned correlation between the anatomy and achieved DVH from prior treatment plans at our institution (as shown in Figure 3). First, anatomical information and DVHs of the PTVs and OARs are extracted from a database of clinical treatment plans. Principal Component Analysis (PCA) is used to characterize the DVH and the anatomy structure information of these existing plans. For a new set of anatomy structures, this algorithm will predict the achievable/ideal DVHs for the bladder and the rectum in the given anatomy (assuming that the PTV coverage remains the same for all plans). These predicted OAR DVHs provide guidelines to judge the conformity of the adapted treatment plan.

### 2.4. Clinical Implementation and Integration of ART

Clinical implementation and integration of ART should balance the efficiency and the quality of the IMRT treatment. Our ART implementation utilizes an automatic plan-selection engine to determine whether repositioning or reoptimization should be used for the specific daily anatomy, with the goal of minimizing the frequency of reoptimization while maintaining uniform CTV coverage and improved OAR sparing. 

**Figure 4:**
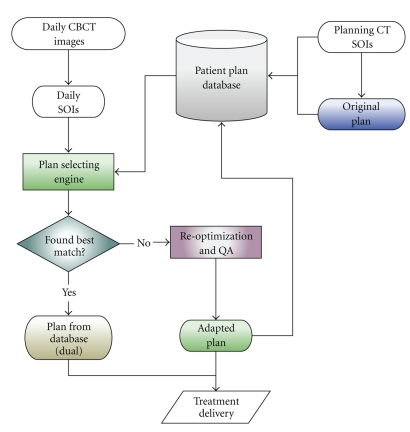
Flowchart of ART implementation. After a new set of daily SOI is acquired, it is compared to existing plans in the database. If an existing plan can be found to match the daily anatomy, it is used for that fraction. Otherwise, an adapted plan is generated via reoptimization for daily treatment and is added to the plan database for future fraction.

The flowchart of the clinical ART implementation is shown in Figure 4. For each patient, a patient-specific plan database is created after the completion of initial treatment planning. Once the daily CBCT is acquired, the CTV and OARs are contoured by the attending physician or via deformable registration. The daily CTV is then compared to the PTVs of all existing plans (1st treatment day only has original CT plan) in this patient's plan database, and the plan that covers >99% daily CTV and minimizes exposure to the OARs is selected as the best fit. If multiple plans meet the criteria, the selection engine then chooses the plan with the smallest PTV. In this way, the selected plan will provide complete CTV coverage and maximize OAR sparing simultaneously.

If no plan in the database can meet the criteria, our replanning process will adapt the initial plan by re-optimizing the fluence maps (as described in Section 2.1). The patient is then treated with the adapted plan for that fraction. After delivery, this adapted plan is added to the plan database for future fractions.

To evaluate the efficacy of the ART scheme, the daily plans from this proposed dual-technique scheme (Dual) are compared with daily reoptimized new plans (New), which represent the most conformal plans for the anatomy of the day, and with the currently used repositioning technique based on soft-tissue target matching plans (Soft). Such comparison is based on the following key dosimetric parameters: dose to 99% CTV volume (D99) and doses to 90% (D90), 50% (D50), and 30% (D30) volumes of the OARs. 

The efficiency of the ART implementation scheme is measured as the reduction of frequency of reoptimization while maintaining the similar CTV coverage and OAR sparing as the daily New plans. Although the dose warping and reoptimization process using our algorithm takes about 2 min, it is a complicated procedure, and the QA takes additional time. The adapted plan also needs approval before delivery. By contrast, existing plans in the patient plan database are approved plans that can be directly reused without significant change in current clinical flow. Therefore, reducing the frequency of reoptimization would generally improve the overall efficiency of ART implementation.

All the dose distributions of the Adapt plans are calculated in PLUNC (Department of Radiation Oncology, University of North Carolina, Chapel Hill, North Carolina), and dose distributions of the New, the Soft, and the Dual plans are calculated in Eclipse. The dose calculation engines in these two systems are different, as PLUNC uses delta-SAR-based method and Eclipse uses pencil-beam convolution method [18]. The discrepancies between dose distributions calculated by these two systems are within 2% for 3D conformal prostate treatment planning. The discrepancies for IMRT are assumed to be of the same order [18]. Difference of dose calculated based on CT and CBCT image might also exist. However, a study at our institution [29] demonstrated that the difference is minimal; the MU/cGy differences were less than 1% for most phantom cases, and the isodose distribution from two calculations agreed very well. Yang et al. also reported that dose computed based on CT and CBCT agreed to within 1% [30]. 

## 3. Results and Discussion

### 3.1. Adapted Plan by Fast Reoptimization Algorithm

**Figure 5:**
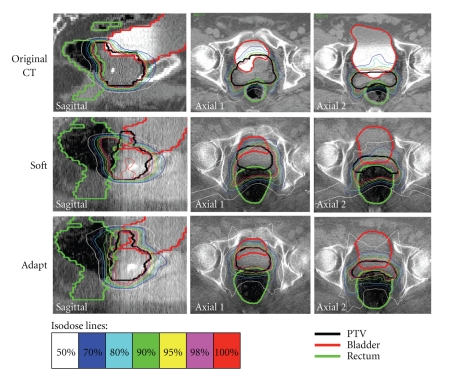
Isodose distribution comparison of the original CT plan (Original CT), the repositioned plan (Soft) and the adapted plan (Adapt) for one treatment fraction. Overlapping of PTV and OARs is caused by the manual expansion from CTV (prostate + rectum) with a 5 mm uniform margin.

Figure 5 shows one example case where daily anatomical variation is significant. The original CT plan, the repositioning only plan (Soft), and the adapted plan (Adapt) are presented. Due to the significant deformation of the bladder (red contours) and the rectum (green contours), the position and volume of daily PTV (black contours) are substantially different from that of the planning PTV on the original CT. Therefore, even after repositioning, the daily PTV suffers significant underdosage in the superior direction, illustrated by part of volume not covered by the 95% isodose line in the Soft plan. In addition, a large portion of rectum is irradiated with high dose in the Soft plan. Improvement of target coverage and OAR sparing is achieved in the Adapt plan, with highly conformal isodose lines to the daily PTV.

The CBCT images used in this study may not have the same image quality, for example, soft tissue contrast, compared to the planning CT; therefore, the contouring on CBCT images is more challenging. To assure consistency, all the structures on both CT and CBCT images studied in this paper were retrospectively contoured by a single experienced attending physician at our institution. We assume the contours in CBCT are consistent with those in the planning CT, as well as between fractions. 

In this example, the relative locations of prostate and SV significantly alter the PTV shape, causing discrepancies between the daily and the planning anatomies. The proposed reoptimization technique is especially beneficial if large deformational variation of organs is present. In clinical practice, such large variation of bladder and/or rectum shape and volumes may sometimes be reduced by patient instructions and additional preparations, for example, treating with a full bladder or emptying rectal gas. The benefit of implementing ART system is to be further validated against such attempt in future work.

### 3.2. Dosimetric Benefit of Adapted Plans over Repositioned Plans

**Figure 6:**
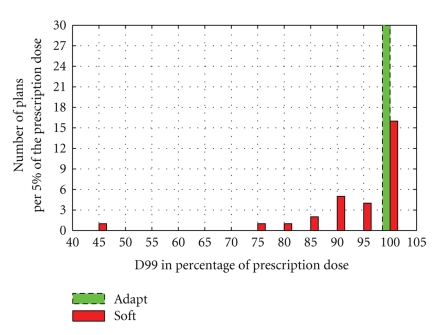
Dosimetric comparison between the on-line reoptimized plan (Adapt) and the repositioned plan (Soft). Shown is the histogram of D99 of CTV for the total 30 CBCT fractions of 6 patients. For each labeled value on the horizontal axis, the corresponding bin extends to ±2.5% from that value.

Figure 6 shows the D99 (minimum dose to CTV) of the daily CTVs for the 6 clinical cases with 30 total treatment fractions. The adapted plans (Adapt) from our fast reoptimization algorithm are evaluated against the repositioning plans (Soft). As shown, the Adapt plans feature highly consistent full CTV coverage, demonstrated by all D99 values ranging within (100 ± 2.5)% of the prescription dose. The daily D99 of the repositioning technique, on the other hand, fluctuates substantially, ranging from 45% to 103%. Overall, the CTV coverage is compromised in 46% of the Soft plans.

**Figure 7:**
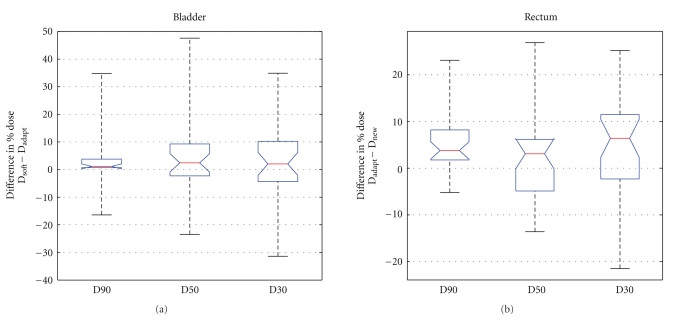
Dosimetric comparison of OAR sparing between the reoptimized (Adapt) and the repositioned (Soft) plans. The figures shown are the differences between the Soft and Adapt plans, that is, the percentage dose differences delivered to each volume indices (90%, 50%, and 30% volume, resp.). Redline: median value; box: 25th ~ 75th percentile range; notch: 95% confidence level; dashed lines: full range of data.

To evaluate OAR sparing between the Adapt and the Soft plans, the percentage dose differences delivered to each volume indices (90%, 50%, and 30% volume, resp.) are computed for the 30 CBCT cases.  Figure 7 shows the statistical analysis between the Adapt and the Soft plans for the D90, D50, and D30 of the bladder and the rectum. Positive values on the *y*-axis suggest that the reoptimization technique has reduced dose compared to the repositioning technique for the corresponding volume, and vice versa. The red lines, blue boxes, and black dashed lines represent the median, 50% range and full range of data in each group, respectively. Notches on boxes indicate the intervals of 95% confidence level. For both bladder and rectum, the median values are all above zero, and majority of data support the assumption that reoptimization technique is superior to repositioning technique, at 95% confidence level. 

Single-side *t*-test reveals that the online ART technique is superior to the soft-tissue-based repositioning technique in low-dose (*P* = .0156) and median-dose (*P* = .0377) ranges for the bladder, and in low-dose (*P* < .0001) and high-dose (*P* = .0247) ranges for the rectum. The advantage of Adapt plans over Soft plans in high-dose range for the bladder and median dose for the rectum is statistically insignificant (*P* = .1318 and  .1672, resp.).

**Figure 8:**
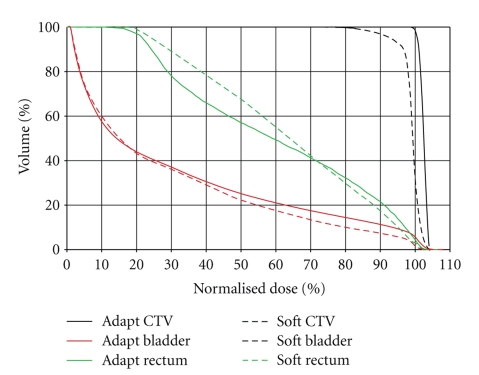
Integrated DVH of an example case where false gain in OAR sparing associated with compromised CTV coverage in the Soft plan.

As seen in Figure 7, for some cases, repositioned plans feature better OAR sparing than adapted plans. However, these improvements are often associated with compromised CTV coverage.  Figure 8 shows an example case. In this case, although the Soft plan provides better sparing for the bladder and the rectum in the high-dose regions, the CTV coverage is significantly compromised, with D99 at 84.6%. Such false OAR sparing with compromised CTV coverage is often seen in cases with large SV shift due to bladder and/or rectum deformation.

The random error in daily CTV coverage could be “washed out” when cumulative dose of all fractions is calculated [31, 32]. In this study, only fractional dose was compared since the focus of our online ART system is to achieve desired dose of each treatment.

### 3.3. Plan Quality Evaluation

**Figure 9:**
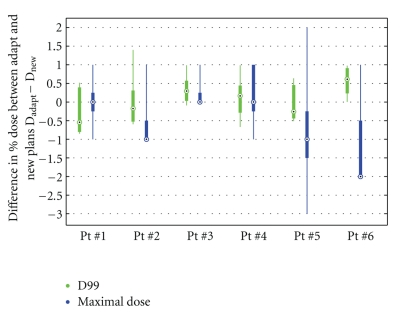
Head-to-head comparison between reoptimized plans (Adapt) and the new plans (New), based on D_max_ (blue, right) and D99 (green, left) of CTV. Dose differences between New and Adapt plans are calculated by subtracting D_New_ from D_Adapt_. Black dots represent the median value of each parameter; thick bars indicate 25th ~ 75th percentile range, while thin lines extend to the full range of all five fractions. For Pt nos. 2, 3, and 6, since the maximal doses for majority of fractions are the same, the dots overlap with the 25% and the lower limit of the sample range.

Head-to-head comparison between the Adapt plans and the New plans is performed for the same 6 patients.  Figure 9 shows the comparisons between the Adapt plans and the corresponding New plans for CTV coverage. Both D99 (green) and D_max_ (blue) values are compared by calculating the difference between the Adapt and the New plans. The *y*-axis indicates the deviation of the Adapt plans from the New plans. If Adapt plans have identical D99 and D_max_ to the New plans, the deviations would be zero. Positive value suggests that the Adapt plan gives higher D99/D_max_ compared to the New plan, whereas negative value indicates that the Adapt plan has lower D99/D_max_. Overall, Adapt plans offer highly comparable CTV coverage to New plans. The deviations for D99 are within −0.3% to 1.4% of the prescription dose, and the deviations for D_max_. range within −3% to 2%, indicating that the capability of controlling low-dose (cold spot) and high-dose (hot spot) regions in the Adapt plans is similar to the New plans. 

**Figure 10:**
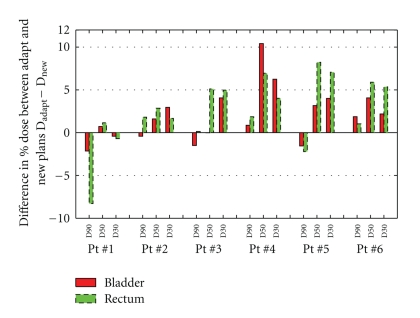
Head-to-head comparison between adapted plans (Adapt) and complete new plans performed with clinical treatment planning system (New), based on averaged D90, D50, and D30 to the bladder and the rectum. *y*-axis indicates the difference in percentage dose between the Adapt and the New plans.

Figure 10 compares OAR sparing between the Adapt and the New plans. The *y*-axis indicates the difference in average D90/D50/D30 between the adapted plans and the new plans. Positive value suggests that the Adapt plans give higher dose to a particular volume than the New plan, whereas negative value indicates better sparing achieved by the Adapt plans. In general, the Adapt plans are slightly inferior to the New plans in the median- and high-dose ranges (D50, D30), demonstrated by positive values being the majority, but in most cases the discrepancies are within 5%, with the rest ranging from 8% to 10%. In low-dose range (D90), most of the Adapt plans are similar to the New plans (within ±2.5%), with one exception in which the Adapt plan shows 8% lower dose than the New plan. 

**Figure 11:**
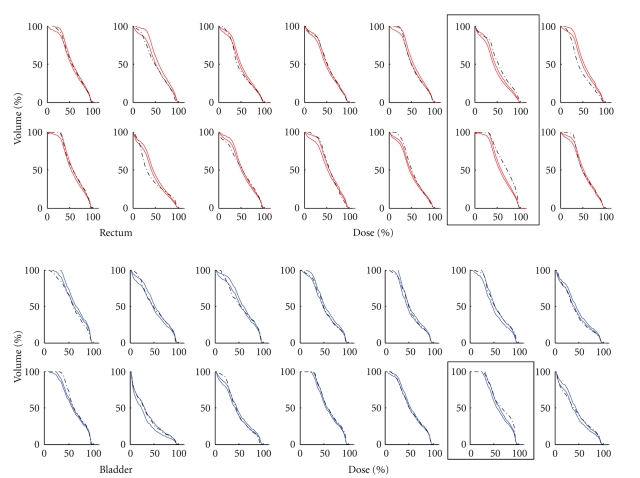
Plan quality evaluation tool for bladder and rectum DVHs. The colored lines (red for rectum and blue for bladder) show the 95% confidence band of the predicted DVH curves, and the black dashed lines show the actually achieved DVHs. Black boxes highlight plans having inferior actual DVHs than predicted.

The plan quality evaluation tool is based on machine learning and is *trained* using a database of previous 198 *expert* plans to establish an anatomy-DVH model. As shown in Figure 11, the evaluation system utilizes the anatomical structure information from input cases and uses the trained model to predict the achievable/ideal DVH bands (colored lines) with 95% confidence levels for 14 test cases. Also shown are the actual structure DVHs from the Adapt plans (dashed black lines). Highlighted by black boxes are plans whose actually achieved DVHs are inferior to the predicted DVHs, indicating possible improvement on plan quality. 

For this proof-of-concept study, the prediction algorithm implemented in this study is learned from a database of plans at our institution and demonstrated to be effective using test cases acquired from the same clinical settings, for example, imaging device, contouring conventions. However, care should be taken when implementing this algorithm at another institution, and the learned model in this study should be tested and validated for the particular clinical settings at that institution. Further, the algorithm can be re-trained to the new plan database of that institution. 

### 3.4. Clinical Implementation and Integration of ART

**Figure 12:**
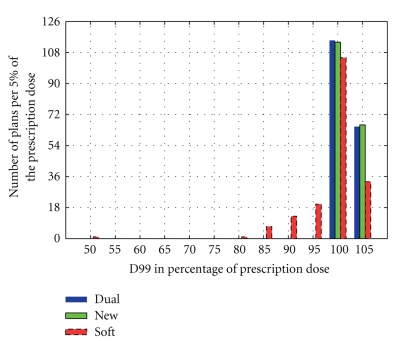
Histogram of 180 plans showing CTV coverage comparison between the dual-technique ART plans (Dual), the repositioning IGRT plans (Soft), and the daily reoptimization plans (New). For each labeled value on the horizontal axis, the corresponding bin extends to ±2.5% from that value. The Bars from left to right in the same D99 bin are Dual, New, and Soft.

Figure 12 shows the histograms of the CTV coverage of the dual-technique ART plans (Dual), the repositioning IGRT plans (Soft), and the daily reoptimization plans (New), respectively. Both the Dual and the New plans exhibit highly uniform CTV coverage; D99 values for all plans are mostly concentrated in the 98% to 105% prescription dose range. 

**Figure 13:**
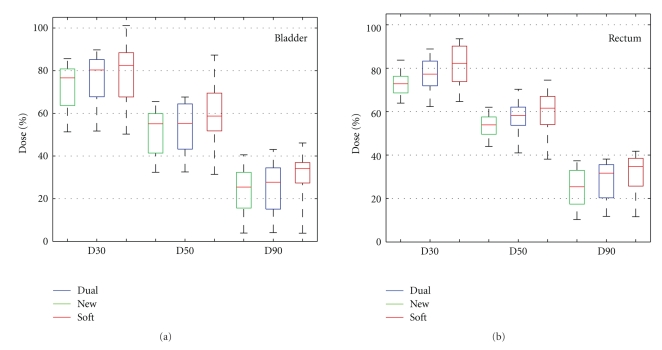
Comparison of OAR sparing for the Dual (middle), the New (a), and the Soft (b) plans for 18 patients (total of 180 plans). Each column indicates the full range (dashed line), 25th ~ 75th percentile range (colored boxes), and the median value (red horizontal lines in each box) of the D30s, D50s, and D90s.

OAR sparing of the Dual plans is also compared against the New and the Soft plans, and the result is illustrated in Figure 13. Quantitative analysis is based on averaged D90, D50, and D30 over 10 CBCT fractions for each patient. Each column in Figure 13 shows the full range (dashed lines), the 25th to 75th percentile range (color boxes), and median value (red lines) of the D30, D50, and D90 values. Compared to the Soft plans, both the New and the Dual plans feature smaller median value and smaller ranges for all three parameters for both organs, indicating better and more consistent OAR sparing performance. The New plans represent the control sample with the most conformal isodose distribution and therefore have best OAR sparing performance. The Dual plans offer bladder sparing comparable to the New plans within 5% variation in both median value and 25th–75th ranges. For the rectum, the Dual plans have less sparing compared with the New plans but still show improvement over the Soft plans. For some patients, the Soft plans have lower dose to the OAR compared to the New plans, as seen in D30 for the bladder and D50 for the rectum, but such apparent sparing is often associated with compromised CTV coverage, as illustrated in Figure 8.

**Figure 14:**
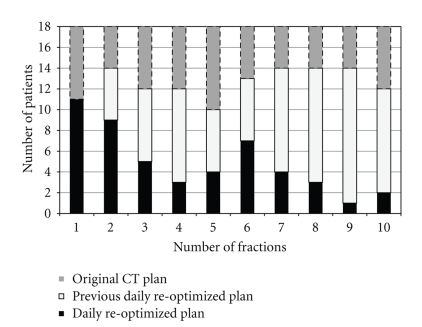
Efficiency of the dual-technique ART implementation. Solid black bars indicate the number of patients treated with reoptimized plans for a particular fraction; light grey bars indicate the number of patients treated with previous daily reoptimized plans, and dark bars indicate the number of patients treated with repositioned original CT plan.

The efficiency of the dual-technique ART implementation scheme is presented in Figure 14. A total of 18 patients' simulated treatment processes are followed and analyzed. Solid black bars are the numbers of patients that are treated with daily reoptimized plans for a particular fraction, due to the unsuccessful matching between the daily structures and the existing plans in the patient-specific database. As more fractions are delivered and more reoptimized plans are cumulated in the patient plan database, the number of necessary replanning patients decreases (shorter black bars), corresponding to the increasing number of patients treated with existing plans in the database, that is, original CT plan (dark grey bars) and previous daily reoptimized plans (light grey bars). The reduction on the need for reoptimization could benefit institutions treating large number of patients at the same time, as reusing existing plans from the database requires minimal change in the current clinical flow. 

### 3.5. Remaining Technical Challenges

This paper provides an overview of different components of our on-going work towards developing an adaptive radiation therapy system. There are still several technical challenges that need to be addressed before this ART system can be clinically implemented. The online reoptimization process has been implemented in PLUNC and CPLEX (IBM Corp.), while currently in the clinical practice at our institution, Eclipse and ARIA (Varian Medical Systems, Palo Alto, CA) are the treatment planning and delivery platforms. To integrate the online reoptimized plan to the clinic, data interfaces between PLUNC-CPLEX and Eclipse-ARIA need to be configured in order to transfer the adapted plans. Also, efficient quality assurance (QA) technique for ART plans needs to be developed to maximize the efficiency of ART. Finally, the evaluation of the benefit of our ART system should be extended to radiobiological indices, including EUD, TCP, and NTCP [18].

## 4. Conclusion

An on-line Adaptive Radiation Therapy (ART) system for prostate cancer is developed to efficiently account for interfractional organ motion/deformation and improve daily target coverage and OAR sparing. Our fast plan reoptimization algorithm produces adapted plans featuring substantially improved daily target coverage and OAR sparing compared to current IGRT technique based on repositioning. The plan quality of reoptimized plans is comparable to those generated by full-fledge inverse planning and optimization in commercial treatment planning system. In addition, a plan quality evaluation tool, predicting achievable DVH based on patient's daily anatomical structure information and learned anatomy-DVH model, is developed to ensure that reoptimized plan meets clinical requirement. Finally, the dual-technique scheme, in which repositioning is expanded to all delivered plans in the patient-specific plan database and integrated with reoptimization technique on a need-based fashion, is clinically feasible, highly efficient, and dosimetrically beneficial. In conclusion, the on-line ART system is technically achievable with deformable registration, fast reoptimization algorithm, and the proper integration of multiple systems.
